# Comments on the article “Social desirability bias in qualitative health research”

**DOI:** 10.11606/s1518-8787.2023057005419

**Published:** 2023-10-30

**Authors:** Maria Lúcia Magalhães Bosi

**Affiliations:** I Universidade Federal do Ceará Faculdade de Medicina Programa de Pós-graduação em Saúde Pública Fortaleza CE Brasil Universidade Federal do Ceará . Faculdade de Medicina . Programa de Pós-graduação em Saúde Pública . Fortaleza , CE , Brasil

One of the main challenges for the community of qualitative health researchers is to be in contact with the hegemony of the positivist paradigm. The absence of social theory and epistemological knowledge in health education, associated with the aforementioned positivist hegemony, causes mistakes to be reproduced that always need to be problematized and clarified. The article in question, object of our comments, published in volume 56 of this renowned journal, intends to discuss what the author considers as a “bias”: social desirability. This is conceived as a “systematic research error,” which can be “identified” and “controlled” in qualitative research, through a set of strategies indicated in the text.

A first element to be highlighted is the absence of “bias” or “systematic errors” in qualitative research. This language belongs to the positivist paradigm; and we know, with Bachelard ^1^ , that the interpretative or constructionist paradigm, to which any qualitative research is inexorably linked, operates an epistemological rupture, a complete split with the canons of the positivist paradigm, for which the data must be objective, independent of the observer and the context. The position of the qualitative approach is the ontological existence of multiple realities, which exist in the form of diverse, historically and socially situated subjective or symbolic productions. In qualitative research, there is no true interpretation. Nor false. When crossing the portal that separates the interpretive paradigm from the positivist one, the first requirement is precisely that of abandoning the positivist notion of truth, in the singular. That which is located somewhere, beyond human experiences and relationships. The results are always constructions negotiated between social actors. Therefore, if there is no truth, the idea of “systematic error” cannot be sustained, even though the validity and fidelity of the results must be guaranteed.

Analyzing what the author considers as “determinants of social desirability bias,” it is interesting to observe the absence of an analysis of the categories power, gender, class, among others that, in an intersectional perspective, would clarify much more rigorously the reasons why a participant responds one way rather than another in a given situation. Or is silent. The analysis of silence is very relevant and the researcher-effect ^2^ has been recognized for some time. Therefore, the attempt to “control” the research situation in the interpretative paradigm, seeking precision and accuracy, illustrates what Prasad ^3^ calls “qualitative positivism.” That is, a reasoning that, despite employing or focusing on non-quantitative methodologies or techniques, maintains positivist rationality, focusing on the mistake of arguing the interpretative paradigm from external and even contradictory criteria, given the aforementioned epistemological rupture. In other words, it makes no sense to consider that the criteria for assessing the quality or sustaining the scientific rigor of the results obtained in interpretative research can be the same as those adopted by quantitative research ^4 , 5^ . This is a mistake that is often observed in the health field, contributing to discredit and to the construction of important obstacles to funding publications, that is, to the scientific and symbolic capital of the community of qualitative researchers.

Finally, it is worth commenting on the “strategies” suggested by the author to “control” the “bias” demarcated for analysis. The content listed in the eight suggestions represents nothing more than a kind of consolidation of good practices for the development of qualitative research, already widely discussed in the available manuals. None of them guarantees the “truth” in the answers. Meaning is constructed, rather than discovered. Finally, it is important to reiterate that the validity and fidelity of qualitative research will only be achieved through reflexivity and onto-epistemological congruence ^6^: operations that demand training and epistemological deepening in the approach, still constituting challenges in the scope of qualitative research in the health field.
