# Supplementary material for: Comments on the article “Social desirability bias in qualitative health research”
Source: Rev Saude Publica. 2023 Oct 30;57:81. doi: 10.11606/s1518-8787.2023057005419 (PMC10631749; doi:10.11606/s1518-8787.2023057005419)
Supplement: Translation

# Translation: Comentários sobre o artigo “Social desirability bias in qualitative health research”

**Authors:** Maria Lúcia Magalhães Bosi

**Affiliations:** I Brasil Universidade Federal do Ceará. Faculdade de Medicina. Programa de Pós-graduação em Saúde Pública. Fortaleza, CE, Brasil

Um dos desafios centrais para a comunidade de pesquisadores qualitativos em saúde é conviver com a hegemonia do paradigma positivista. A ausência de teoria social e conhecimentos epistemológicos nas formações em saúde, associada à referida hegemonia positivista, faz com que se reproduzam equívocos que precisam ser sempre problematizados e esclarecidos. O artigo em tela, objeto dos nossos comentários, publicado no volume 56 deste renomado periódico, pretende discutir o que o autor considera um “viés”: a desejabilidade social. Esta é concebida como “erro sistemático de pesquisa”, passível de ser “identificada” e “controlada” nas pesquisas qualitativas, mediante um conjunto de estratégias apontadas no texto.

Um primeiro elemento a ser sinalizado é a inexistência de “vieses” ou “erros sistemáticos” na pesquisa qualitativa. Essa linguagem pertence ao paradigma positivista; e sabemos, com Bachelard ^1^ , que o paradigma interpretativo ou construcionista, ao qual inexoravelmente se vincula qualquer pesquisa qualitativa, opera uma ruptura epistemológica, uma cisão completa com os cânones do paradigma positivista, para o qual os dados devem ser objetivos, independentes do observador e do contexto. A posição do enfoque qualitativo é a existência ontológica de múltiplas realidades, que existem sob a forma de produções subjetivas ou simbólicas diversas, histórica e socialmente situadas. Na pesquisa qualitativa, não há interpretação verdadeira. Nem falsa. Ao cruzar o portal que separa o paradigma interpretativo do positivista, a primeira exigência é precisamente a de abandonar a noção positivista de verdade, no singular. Aquela que se situa em algum lugar, para além das experiências e relações humanas. Os resultados são sempre construções negociadas entre atores sociais. Portanto, não existindo verdade, a ideia de “erro sistemático” não se sustenta, ainda que se deva garantir a validade e a fidelidade dos resultados.

Analisando o que o autor considera como “determinantes do viés de desejabilidade social”, é interessante observar a ausência de uma análise das categorias poder, gênero, classe, dentre outras que, numa perspectiva interseccional, esclareceriam de forma bem mais rigorosa os motivos pelos quais um participante responde de uma forma e não de outra numa dada situação. Ou silencia. A analítica do silêncio é muito relevante e há tempos se reconhece o efeito-pesquisador ^2^ . Portanto, a tentativa de “controlar” a situação de pesquisa no paradigma interpretativo, buscando precisão e exatidão, ilustra o que Prasad ^3^ denomina “positivismo qualitativo”. Ou seja, um raciocínio que, não obstante empregue ou focalize metodologias ou técnicas não quantitativas, mantém a racionalidade positivista, incidindo no equívoco de arguir o paradigma interpretativo a partir de critérios externos e mesmo contraditórios com ele, haja vista a aludida ruptura epistemológica. Noutros termos, não faz sentido considerar que os critérios para aferição da qualidade ou sustentação do rigor científico dos resultados obtidos na pesquisa interpretativa possam ser os mesmos que os adotados pela pesquisa quantitativa ^4 , 5^ . Esse é um equívoco que se observa seguidamente no campo da saúde, contribuindo para o descrédito e para a construção de obstáculos importantes para o financiamento, publicações, ou seja, para o capital científico e simbólico da comunidade de pesquisadores qualitativos.

Por fim, cabe comentar as “estratégias” sugeridas pelo autor para “controlar” o “viés” demarcado para análise. O conteúdo recenseado nas oito sugestões representa nada mais que uma espécie de consolidado de boas práticas para o desenvolvimento de pesquisas qualitativas, já amplamente discutidas nos manuais disponíveis. Nenhuma delas assegura a “verdade” nas respostas. O significado é construído, e não descoberto. Finalizando, é importante reiterar que a validade e fidelidade das pesquisas qualitativas somente serão alcançadas pela reflexividade e pela congruência ontoepistemológica ^6^: operações que demandam formação e aprofundamento epistemológico no enfoque, constituindo ainda desafios no escopo das pesquisas qualitativas no campo da saúde.
